# Erratum: Identification of hub genes and construction of prognostic nomogram for patients with Wilms tumors

**DOI:** 10.3389/fonc.2023.1136978

**Published:** 2023-01-18

**Authors:** Frontiers Production Office

**Affiliations:** Frontiers Media SA, Lausanne, Switzerland

**Keywords:** wilms tumor, hub gene, risk stratification, prognostic model, nomogram

Due to a production error, an author name was incorrectly spelled as “Lie Lou”. The correct spelling is “Lei Lou”. The publisher apologizes for this mistake.

The original version of this article has been updated.

